# Metal Attraction: An Ironclad Solution to Arsenic Contamination?

**DOI:** 10.1289/ehp.113-a398

**Published:** 2005-06

**Authors:** Lance Frazer

## Abstract

Inorganic arsenic—the more acutely toxic form of this metalloid element—contaminates drinking water supplies around the world. In the United States, the most serious arsenic contamination occurs in the West, Midwest, Southwest, and Northeast; as many as 20 million people—many getting their water from unregulated private wells—may be exposed to excess arsenic in their drinking water. In Bangladesh, it’s estimated that as many as 40 million people may be suffering from arsenic poisoning; contaminated drinking water is also a problem in many other countries, including Argentina, China, Chile, Ghana, Hungary, India, and Mexico.

There are several methods for removing inorganic arsenic from water. Many take advantage of the strong bond that forms between arsenic and iron. Now Littleton, Colorado–based ADA Technologies, through funding from the NIEHS Superfund Basic Research Program, the U.S. Air Force, the U.S. Environmental Protection Agency (EPA), and the state of Colorado, has gone a step farther in capitalizing on that characteristic with a new class of amended silicate sorbents that remove even more arsenic from water, and do it more easily and more cheaply. ADA is also working with researchers at Virginia Polytechnic Institute and State University and Old Dominion University to study the interaction between arsenic species and iron oxide–based media, and is collaborating with other partners to develop low-cost approaches for quantifying the concentration of arsenic in drinking water.

## A Host of Health Risks

Most environmental arsenic occurs naturally, appearing in deposits of minerals and ores including arsenopyrite, enargite, and proustite. A smaller but still significant source of arsenic exposure is anthropogenic. Inorganic arsenic as well as various arsenical compounds have been used in agricultural chemicals and wood preservatives, in the glass industry, and in the production of lead shot. Elsewhere, emissions from coal-burning power plants are a significant source of arsenic exposure.

However, the majority of toxic exposure comes from drinking water contaminated with naturally occuring arsenic. Chronic arsenic ingestion through drinking water is known to cause skin cancer, and has been linked to an increased risk for cancers of the bladder, lung, kidney, liver, colon, stomach, uterus, and prostate. Arsenic has also been associated with cardiac, pulmonary, and artery diseases, diabetes mellitus, and neurological, developmental, and reproductive problems.

In the United States, a revised drinking water standard for arsenic of 10 micrograms per liter (μg/L) is set to take effect in January 2006, but there is substantial concern that this level is still too high for public safety. In many other countries, allowable levels are even higher. With millions of people around the world facing potential adverse health effects from this contaminant, the need for effective, affordable ways to remove arsenic from drinking water is critical.

## Ridding Water of Arsenic

Arsenic is generally found in two inorganic forms in nature—arsenate and arsenite. Arsenate is present as a negatively charged ion at typical drinking water pH (roughly 6.5–8.5), whereas arsenite is neutral in the same pH range. Many treatment methods rely on a negative arsenic charge, so they tend to be more successful at capturing arsenate.

One such method is ion exchange, which uses polystyrene-based resins containing positively charged sites to remove negatively charged species. Besides being effective only with arsenate, sulfate ions are removed preferentially to arsenic, so if large amounts of sulfate are present, those ions will tie up the bonding sites, leaving fewer available for arsenic to bond with. Activated alumina is a filter medium that will remove a variety of contaminants, including fluoride, arsenic (both arsenite and arsenate), and selenium, but it requires periodic cleaning with an appropriate regenerant such as alum or caustic in order to remain effective. Activated alumina also is effective only across a very narrow pH range (6 to 7).

Granular ferric oxide is an iron-based adsorbent that can capture both arsenate and arsenite, but in general, it functions best at or below a pH of 7, and both phosphates and silicates can interfere with its action. A fourth method, a coagulation/filtration process, uses a ferric chloride liquid and an oxidizing agent such as sodium hypochlorite to create insoluble ferric hydroxide. Arsenic adsorbs readily onto the solids, but workers must store and handle corrosive ferric oxide and oxidant solutions.

The ADA formulation takes a slightly different approach. The basic ingredient is an iron oxide known as akageneite. According to Craig Turchi, ADA program manager for the arsenic project, the company focused on an iron oxide because iron tends to form very strong, stable bonds with arsenic. Additionally, Turchi explains, oxides tend to be among the most stable substances in nature, and many of them tend to accumulate the substances with which they come into contact, including many contaminants. “Iron oxide is a molecule with hydroxyl groups,” he says. “And we know that, from a chemical perspective, arsenic behaves like a hydroxyl in some ways.”

One of the key advantages to ADA’s approach is that the akageneite is coated onto an inert silicate substrate. In comparison, most other approaches involve pure iron oxide granules. Turchi says, “Our tests have shown we can get the same capacity as our competitors, but with much less iron. And that cuts the cost from around four dollars per pound down to around two dollars per pound.” Additionally, because ADA’s akageneite particles are on the nanoscale, they can be dispersed far more efficiently in water undergoing treatment. The ADA formulation also is notable for its ability to remove both arsenite and arsenate, and its effectiveness at a wider pH range of 6.5–8.5.

## A Super Sorbent

The ADA formulation has been shown to reduce arsenic contamination as high as 1,000 μg/L to 10 μg/L in as little as 30 minutes. “The capacity of the adsorbent increases with the concentration of arsenic in the water,” Turchi says. “This behavior is typical of most adsorbents.” Capacity varies somewhat with the water content, says Turchi, but generally ADA’s material removes about 2 milligrams of arsenic per gram of sorbent at a concentration of 50 μg/L, and about 40 milligrams of arsenic per gram of sorbent at 1,000 μg/L.

There is a catch, though—as the water gets cleaner and more bonding sites are taken up by arsenic, it becomes harder and harder to remove the last bits of arsenic. Turchi says it probably is not possible to remove all the arsenic from water with processes such as this. “It comes down to the efficiency of an equilibrium process,” he says. “Arsenic has an affinity to stay in water, as well as an affinity to attach to materials like iron. You’ll get diminishing returns until these affinities balance at some point.”

Turchi says users will eventually be able to choose from either a solubilized form of the sorbent that can be sprinkled into contaminated water, circulated, and then filtered off or allowed to settle out, or a pelletized form for use in a packed-bed approach. A packed bed consists of layers of adsorbent material. Contaminated water is poured in the top, and purified water is collected at the bottom after seeping through the material. “In the ideal case you periodically add new sorbent to the clean water end and remove the arsenic-saturated sorbent from the other end, so that you get the maximum use of your sorbent,” Turchi says.

Once the binding sites on the iron oxide have been used up, the material could be reused by acidifying it to break the arsenic–iron bonds, then filtering off the arsenic. But Turchi says economics—and the logistics of dealing with the arsenic-laden, high-pH waste—will probably dictate disposal of the used adsorbent. The spent material has passed EPA tests to determine the likelihood of contaminants leaching out of landfills into the surrounding water supply, so Turchi says the sorbent can simply be disposed of in a regular landfill.

Turchi says ADA’s sorbent system has the benefits of being both robust and simple: no moving parts, and little training required. “I think that makes our system more appropriate for smaller-scale uses,” he says. “Typically, in a large municipal facility, you’d run the water through the filtration system, but a small village [in a developing nation] probably doesn’t have a water treatment facility.”

ADA and collaborator Kinetico Incorporated, a water treatment system engineering company, are planning a field test involving a packed column of the amended silicate for summer 2005 at a facility in New Mexico, where the ADA formulation will be tested against two commercially available competitors. The formulation also underwent earlier field tests at two Colorado sites.

## Questions of Stability

Though not familiar with ADA’s work specifically, Joshua Hamilton, director of the Center for Environmental Health Sciences at Dartmouth College and the Dartmouth Toxic Metals Research Program, says, “I know a lot of people are working with iron oxides, and it appears to be a very fruitful area. Iron oxides and arsenic exhibit tight bonding properties, and oxides are relatively cheap materials. All of these are pluses—high efficiency, low maintenance, low cost, and easily renewable.”

Still, says Hamilton, there are a few aspects of the ADA approach that may be cause for concern. For one, he says, “I think that assuming you can safely put it in a conventional landfill might be overstating the strength of the bond.” He explains that a lot of arsenic is found in granitic deposits throughout his home state of New Hampshire. “We’re seeing, under normal environmental circumstances, the mobilization of a good deal of arsenic out of materials that are basically compounds of arsenic and iron,” he says. “And it should also be taken into account that there’s a lot of interesting and somewhat unpredictable chemistry that goes on in landfills.” He points out that the environmental conditions that allow organics to remain contained are quite different from those that allow arsenic to be contained. “So if you focus on arsenic,” he says, “you could end up releasing organics into the environment and vice versa.”

Turchi agrees that the issue of long-term sorbent stability in landfills needs to be addressed. He explains that the EPA leach tests examine the stability of landfilled contaminants under acidic conditions, because most metals leach off of sorbents under acidic conditions. “Research indicates that arsenic could conceivably leach off the media under alkaline conditions, so a different test may be required,” he says.

Michael Harbut, chief of the Center for Occupational and Environmental Medicine at Wayne State University, points to another concern: “I’d [like] to see enough studies to show me the substance that resulted when the arsenic bonded didn’t have toxic properties of its own—inhalational studies, cardiac trend studies, the whole suite. Then we’d move on to worry about bond strength in the environment.” Harbut has studied low-level arsenic poisoning for many years and has lobbied for stricter water thresholds and broader testing.

Hamilton raises a third concern: the fact that even $2 per pound could prove to be an insurmountable barrier in many countries. With comparable technologies costing $4–8 per pound, ADA’s price does seem a bargain. But with the annual per capita income in Bangladesh, for example, hovering around US$360, the outlay of even $2 per pound of sorbent (enough to remediate about 800,000 liters of water) could well prove prohibitively high.

“One factor that could mitigate that cost would be to make the sorbent material in the country where it will be used,” says Turchi. “Using less expensive labor and avoiding the costs of transportation could lower the cost significantly.”

In the meantime, even affluent countries such as the United States are still searching for an effective, affordable response to arsenic. “You can put a reverse osmosis filter on your sink at a cost of six to eight hundred dollars,” Harbut says. “That’s not much to some, but for too many in this country, that’s just more than they can afford. We need, as a society, to fund these systems for those who can’t afford them.” Municipal water systems appear to have the technology to address the arsenic issue, but private well owners, as well as developing nations whose populations are scattered far beyond the reach of any centralized system, need a fast, safe, reliable system for removing arsenic from water. Further testing will tell if ADA’s amended silicate technology can provide one answer.

## Other Arrows in the Arsenic Arsenal

Many other researchers are seeking the magic combination of a cheap and effective arsenic remediation process. All too often, if a treatment process is effective, it’s not cheap, and if it’s cheap, it’s not effective. A couple of other new ideas being tested might, like the ADA strategy, meet both goals.

Ashok Gadgil, a researcher at Lawrence Berkeley National Laboratory, is working with a by-product of coal burning called bottom ash. Bottom ash (which differs from fly ash in that the former contains no heavy metals) is an ultrafine substance, with particles one-tenth to one-hundredth the width of a human hair. Gadgil and his team coat the ash particles with ferric hydroxide, which in turn bonds powerfully with available arsenic. Initial laboratory tests indicate the substance can reduce arsenic concentrations from 2,400 μg/L to only 10 μg/L within an hour.

Gadgil envisions loading this material into a teabag-sized filter to go in a water jug, providing a Bangladeshi family of six with a day’s safe drinking water. Costs, he estimates, might run around 30¢ per person per year. Gadgil is also testing the material for possible use in a water treatment system for small U.S. municipal water treatment facilities.

On the opposite coast, an engineering team under the direction of Massachusetts Institute of Technology engineering professor Susan Murcott has hit on the idea of a filtration system utilizing layers of sand, brick chips, gravel, and iron nails. Once again, the strong attraction between arsenic and iron comes into play, as tests indicate arsenic contamination can be reduced to 10 μg/L within an hour. Cost of the initial system is about US$16 per year.

Only time—and much more testing—will tell whether these approaches or any of the others being developed around the world will meet all of the criteria of simplicity, reliability, and ease of use. One additional incentive to find such an approach is the new Grainger Challenge Prize for Sustainability. The National Academy of Engineering is offering this $1 million prize to help solve the massive public health problem of arsenic contamination. The prize will be awarded to an individual or group for the design and creation of a workable, sustainable, economical point-of-use water treatment system for arsenic-contaminated groundwater in Bangladesh, India, Nepal, and other developing countries. The first Grainger Challenge prize will be awarded in February 2007.

## Figures and Tables

**Figure:**
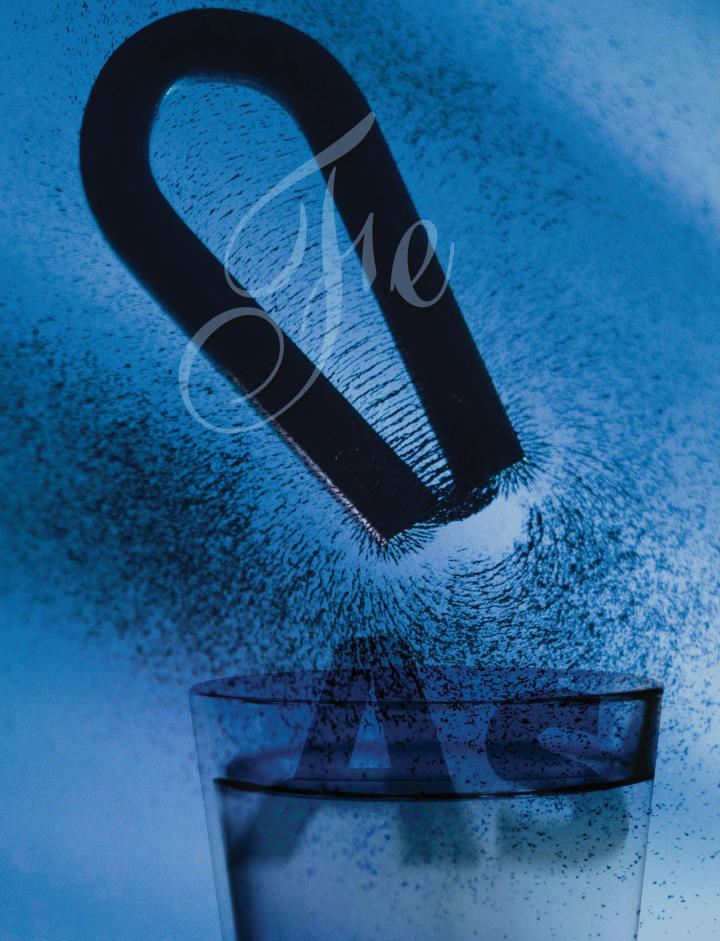


**Figure:**
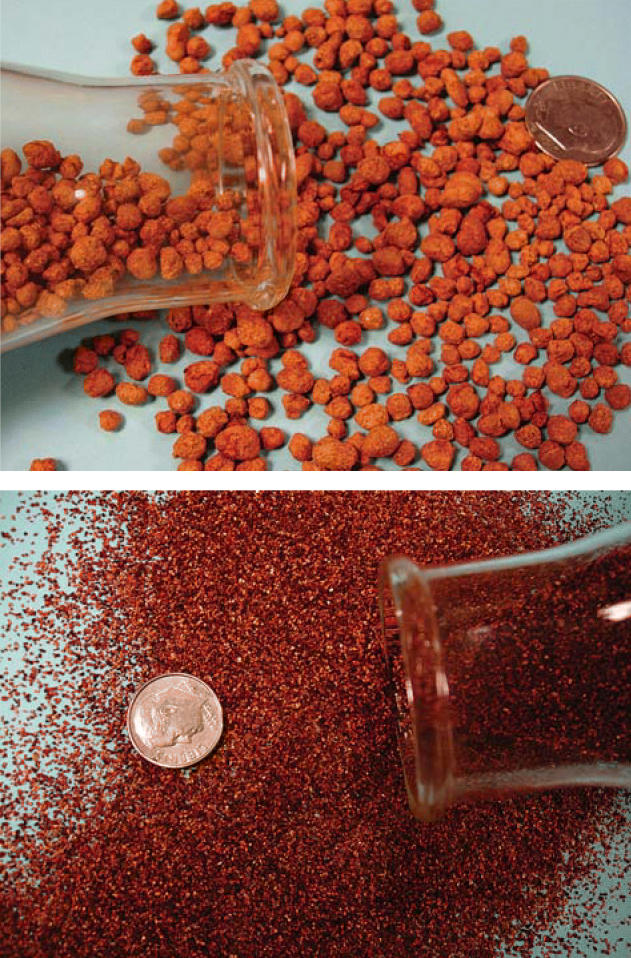
**The stuff of sorbency.** ADA’s amended silicate sorbent comes in two formulations, one more suitable for a packed-bed approach (top) and a finer version that can be sprinkled into water, then filtered off (bottom).

**Figure:**
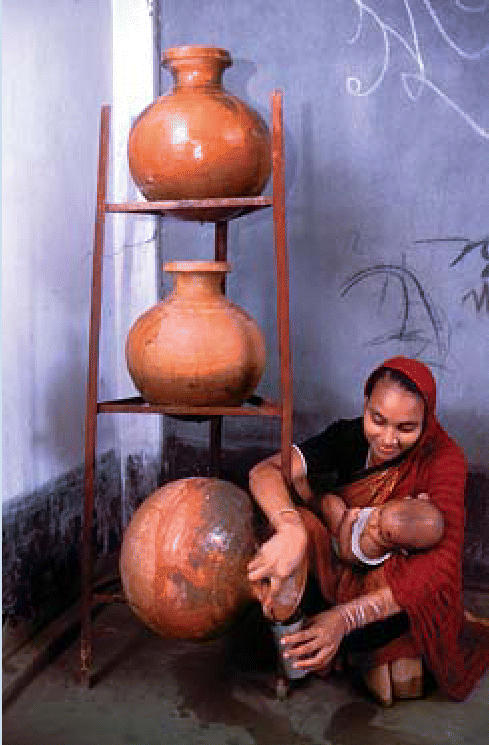
**Juggling strategies.** Simple arsenic mitigation methods that can be used at home are one target of ongoing research.
